# Membrane Separation of Chicken Byproduct Hydrolysate for Up-Concentration of Bioactive Peptides

**DOI:** 10.3390/membranes14020028

**Published:** 2024-01-23

**Authors:** Janka Dibdiakova, Josipa Matic, Sileshi Gizachew Wubshet, Wolfgang Uhl, Lelum Duminda Manamperuma, Bjørn Rusten, Eilen Arctander Vik

**Affiliations:** 1 Aquateam COWI AS, Karvesvingen 2, 0579 Oslo, Norway; woul@cowi.com (W.U.); mnmn@cowi.com (L.D.M.); bru@aquateam.no (B.R.); eav@cowi.com (E.A.V.); 2 Nofima AS, Osloveien 1, 1433 Ås, Norway; josipa.matic@nofima.no (J.M.); sileshi.wubshet@nofima.no (S.G.W.)

**Keywords:** bioactive peptides, enzymatic protein hydrolysis, membrane filtration, purification, LC-OCD

## Abstract

Membrane processes, such as microfiltration, ultrafiltration, and nanofiltration, are increasingly used for various applications in both upstream and downstream processing. Membrane-based processes play a critical role in the field of separation/purification of biotechnological products, including protein production/purification. The possibility of using membranes to separate peptides from a chicken byproduct hydrolysate and the effect of the performed downstream processing on the DPP-IV dipeptidyl peptidase IV (DPP-IV) inhibitory activity of mechanical deboning chicken residue (MDCR) has been investigated. The chicken byproduct hydrolysate was prepared by enzymatic hydrolysis followed by microfiltration (MF), ultrafiltration (UF), nanofiltration (NF), and reverse osmosis (RO) separation. Comparing all separation treatments, hydrolysates processed only by MF and UF show the best DPP-IV inhibition (59.5–60.0% at 1 mg/mL and 34.2–40.7% at 0.5 mg/mL). These samples show dose-responsive behavior. Bioactivity was correlated with molecular weight distribution profiles and average molecular weights. The nanofiltration process notably decrease the inhibitory activity, and these permeates show low DPP-IV inhibition (9.5–21.8% at 1 mg/mL and 3.6–12.1% at 0.5 mg/mL). The size-exclusion chromatography–organic carbon detection–organic nitrogen detection (LC–OCD–OND) analysis confirms that NF and RO would retain the bioactive peptides in the concentrate in comparison to MF and UF. Bioactivity was correlated with molecular weight distribution profiles and average molecular weights. Permeates after ultrafiltration show an IC_50_ value of 0.75 mg/mL, comparable to other potent DPP-IV inhibitors derived from various food sources, and significantly more potent compared to the microfiltration sample, which shows an IC_50_ value of 1.04 mg/mL. The average molecular weight of the permeates calculated from the SEC chromatograms was 883 g/mol for UF and 1437 g/mol for MF. Of the four membranes studied, the UF membrane shows the best separation properties with respect to maximizing the yield and up-concentration of the bioactive peptides. Overall, UF was demonstrated to be a feasible technology for the removal of the undesired high-molecular-weight substances and up-concentration of small-molecular-weight bioactive peptides from chicken byproduct hydrolysate. These peptides might exhibit biological activity and could offer several health benefits. There is a high potential for the use of bioactive peptides, and more research in this field can lead to promising results that have significant effects in the food and medical industries.

## 1. Introduction

Biotransformation of underutilized protein biomasses (e.g., food processing byproducts) to value-added ingredients is an important element in efficient resource utilization. Enzymatic protein hydrolysis (EPH) is one of the attractive biotechnological processes used for the degradation of protein biomasses, thereby enabling the recovery of valuable ingredients such as bioactive peptides, lipids, and minerals [1,2]. Small-molecular-weight bioactive peptides generated using EPH have great potential as health-promoting ingredients in the form of functional foods and nutraceuticals [3]. Beyond their role as sources of nutrients, such peptides have been shown to have a wide range of biological functions, including blood glucose regulation, and antihypertensive and cholesterol-lowering effects [4,5]. In the production of such bioactive peptides for human consumption, downstream separation technologies are essential unit operations implemented to improve vital sensory attributes as well as to concentrate bioactive constituents [6]. Typically, EPH relies on the use of food-grade protease cocktails with limited specificity and the resulting hydrolysates are complex mixtures of various peptides, undigested proteins, free amino acids, minerals, and other metabolites. Properties of hydrolysates depend both on the raw material used as well as on the process conditions in which they are produced. Specificity of the protease can be altered in different reaction conditions, such as concentration, temperature, or pH. In addition, the substrate availability varies with reaction time. A combination of these factors results in a multitude of peptides being produced, differing in sequences and sizes. Hence, when a specific type of health-promoting bioactive peptide is the aim, it is necessary to have adequate downstream processing technologies (i.e., filtration). Such filtration can have a dual aim of disregarding undesired constituents (e.g., allergenic large proteins) as well as up-concentration of the desired constituents (e.g., bioactive peptides). Bioactivity of a given peptide depends on various characteristics, including amino acid sequences, molecular weight (MW), hydrophobicity, charge, and acid–basic character [7]. Peptides MW has been demonstrated to have a great impact on the bioactivity of protein hydrolysates [8,9]. Lima et al. have shown that small-molecular-weight peptides isolated from chicken byproduct hydrolysates using size exclusion chromatography are associated with in vitro antidiabetic activity [10]. When the molecular weight is given as the determinant factor for a given bioactivity, pressure-driven membrane separations can be used as a downstream unit operation to increase their specific activity.

Membrane separations, including microfiltration (MF), ultrafiltration (UF), nanofiltration (NF), and reverse osmosis (RO), are useful techniques to extract, concentrate, purify, or fractionate valuable molecules from effluents, wastes, or by-products from food processing industries [11,12]. They have been used for a couple of decades to treat protein hydrolysates of dairy origin [13,14], and animal [15] or vegetable/fruit products [16]. It can also be used for protein separation, but it is limited to the isolation of proteins with relatively close molecular weights [17]. It relies on the action of pressure to transport the solvent (usually water) and dissolved substances smaller than the pores through the membrane, while dissolved substances bigger than the pore size and suspended substances are retained. Due to the concentration difference between the two sides of the membrane, osmotic pressure, which adds to the flow resistance of the membrane, needs to be overcome as well [18]. Membrane fouling, which is mainly the adsorption and deposition of retained substances on the membrane surface and subsurface, is the main factor in the process of protein ultrafiltration [19]. According to their molecular weight cut-off (MWCO), UF or NF have been suggested for the separation of peptide hydrolysates [20]. NF can be used to concentrate hydrolysates [21,22], whereas UF membranes with high MWCO (20 to 100 kDa) are adapted to the separation of peptides and non-hydrolyzed proteins or proteolytic enzymes [23,24]. On the other hand, UF membranes with intermediate MWCO (about 4000 to 8000 Da) allow hydrolysates to be fractionated with the result of enrichment in some ranges of molecular weight [25].

This work aimed to study the different filtration processes for the up-concentration of dipeptidyl peptidase IV (DPP-IV)-inhibiting bioactive peptides from chicken byproduct protein hydrolysates. DPP-IV is an important therapeutic target for type 2 diabetes (T2D), and peptides from EPH of byproducts have been indicated as potential inhibitors of this target. The risks of T2D complication are mainly linked to an increase in hemoglobin A1c (HbA1c) [26]. Hence, control of blood glucose level (A1c) constitutes one of the major therapeutic strategies in management of T2D. Incretins (glucose-dependent insulinotropic peptide (GIP) and glucagon-like peptide-1 (GLP-1)) are vital elements in regulation of blood glucose levels, responsible for up to 60% of the insulin response to a given glucose load [27]. A serine protease enzyme, dipeptidyl peptidase IV (DPP-IV), degrades GLP-1 and GIP and drastically reduces the half-lives of these important hormones. Hence, selective DPP-IV inhibitors can prolong the vital role of incretins in glucose homeostasis. An alternative to pharmaceuticals, bioactive peptides have been proposed as a substitute for DPP-IV inhibitors, which can be used for management of HbA1c in the form of nutraceuticals or functional foods [26]. A recent study by Wubshet et al. [8] demonstrates that DPP-IV inhibition of the crude chicken byproduct hydrolysate is associated with low-molecular-weight peptides with an average molecular weight of 500 Da.

Recent scientific evidence suggests that food proteins not only serve as nutrients but can also modulate the body’s physiological functions. These physiological functions are primarily regulated by some peptides that are encrypted in the native protein sequences [28,29]. These bioactive peptides can exert health beneficial properties and, thus, are considered as a lead compound for the development of nutraceuticals or functional foods. The several bioactivities typical for peptides isolated from food proteins include the following: antihypertensive; antimicrobial; antioxidant; antitumor; immunomodulatory activity; binders and mineral ion carriers; hypocholesterolemic; anti-inflammatory; multifunction activity. Among the main food sources of bioactive peptides that should be highlighted are the milk proteins (caseins and whey proteins), egg proteins, fish proteins, meat proteins (cattle, pigs, poultry), and some proteins of cereal grains and legumes [30]. In the past few decades, a wide range of food-derived bioactive peptide sequences have been identified, with multiple health-beneficial activities [29]. However, the commercial application of these bioactive peptides has been delayed because of the absence of appropriate and scalable production methods, proper exploration of the mechanisms of action, high gastro-intestinal digestibility, variable absorption rate, and the lack of well-designed clinical trials to provide the substantial evidence for potential health claims [31,32]. Further studies are required for the future use of food-derived bioactive peptides for the prevention and management of chronic diseases. As there are many factors that may influence the production of bioactive peptides, there is still a need to develop a more scalable, affordable, and consistent production technique.

The present study was designed to evaluate MF, UF, NF, and RO as scalable downstream processes for up-concentration of the low-molecular-weight bioactive peptides and investigate the possibility of reusing their permeates. Experiments of all filtration processes were performed in a laboratory unit, which was operated in a dead-end separation pattern. The main reason for choosing MF, UF, NF, and RO separation processes for this research was the limited amount of the raw chicken byproduct hydrolysate. We were able to produce only around 200 mL of raw hydrolysate and this amount was not sufficient enough to run bigger separation units such as cross-flow. This study did help us to design a much better, efficient, and feasible separation system, which will be the topic of our next scientific work. In this study, the chicken byproduct hydrolysate was fractionated systematically in a four-step process consisting of MF, UF, NF, and RO. To evaluate the performance of the separation processes, the behavior of the membrane permeability during the separation process was obtained from the measurement of mass flow rates. The composition of the chicken byproduct hydrolysate and the separation resulting from the MF, UF, NF, and RO were characterized by size-exclusion chromatography (SEC) and size-exclusion chromatography–organic carbon detection–organic nitrogen detection (LC–OCD–OND) chromatograms. Moreover, in vitro DPP-IV inhibition of the permeates after the different filtration stages were evaluated to guide and optimize the up-concentration of the bioactive peptides. The enzymatic protein hydrolysis hydrolysate has bioactive properties, and it can be produced and purified easily in the laboratory scale. However, for the upscaling, the purification and industrial optimization is not straightforward process. It should be properly developed, and this is a motivation and foundation of this scientific work with the designed process.

## 2. Materials and Methods

### 2.1. Preparation of Chicken Byproduct Hydrolysate

Mechanical deboning chicken residue (MDCR) biomass was supplied by a Norwegian food producer (Nortura, Hærland, Norway). The biomass was homogenized using a food processor, vacuum packed into plastic bags, and stored at −20 °C before the hydrolysis. Hydrolysis of MDCR was performed as described in [8]. The hydrolysis was performed in a Reactor-Ready™ jacketed reaction vessel (Radleys, Saffron Walden, Essex, UK). Water running through the vessel jacket was kept at 60 °C and delivered using a JULABO circulator pump (Julabo GmbH, Seelbach, Germany). Homogenized MDCR (500 g) was suspended in 1 L distilled water and stirred at 300 rpm until the suspension reached 60 ± 1 °C (45 min). Then, the selected protease preparation (FoodPro^®^ PNL, 25 g, 5% *w*/*w*) was added to the stirring mixture to start the hydrolysis. Hydrolysis time was 45 min, after which hydrolysis was quenched by thermal inactivation of the protease. Thermal inactivation was achieved by the rapid increase in temperature in the microwave oven (Menumaster, ACP, Cedar Rapids, IA, USA) followed by heating the mixture at 95 °C for 15 min inside the water bath (Precision GP 10, Thermo Scientific, Waltham, MA, USA). After inactivation, the mixture was centrifugated for 15 min, at 5346× *g* and at 25 °C, using Multifuge4 KR centrifuge (Thermo Scientific, MA, USA) to separate the sediment from the supernatant. The supernatant was transferred into an extraction funnel to separate the oil from the aqueous phase. The aqueous phase was then filtered using Pall^®^ Depth Filter Sheets T2600. The water phase was aliquoted in 250 mL plastic containers and stored frozen at −40 °C, until it was lyophilized using a Gamma 1–16 LSC plus freeze dryer (Martin Christ Gefriertrocknungsanlagen, Osterode am Harz, Germany). The chicken hydrolysate solution used in filtration experiments was prepared by mixing 3.75 g of raw chicken byproduct hydrolysate (RCH) powder in 1 L of deionized water. The amount of 500 mL of the initial solution was divided into 4 filtration series as follows: RCH1 (100 mL of initial solution), RCH2 (80 mL of initial solution), RCH3 (60 mL of initial solution), RCH4 (40 mL of initial solution).

### 2.2. Dead-End Membrane Filtration

#### 2.2.1. Stirred Cell Membrane Filtration Unit

**Figure 1:**
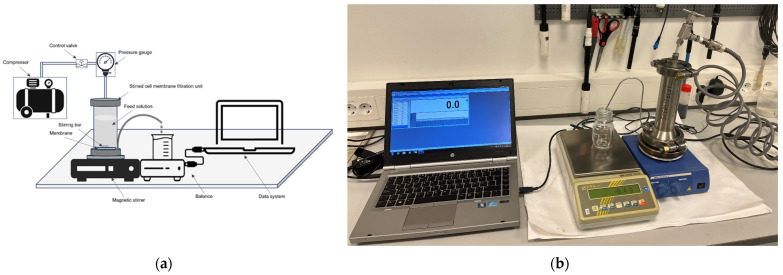
Experiment setup: (**a**) scheme of the experimental setup of the stirred cell dead-end membrane filtration; (**b**) laboratory setup of the stirred cell dead-end membrane filtration of the chicken byproduct hydrolysate.

The setup of the stirred cell membrane filtration unit is shown in Figure 1. Membrane filtration experiments were carried out at an ambient temperature of 19–22 °C, using a high-pressure stirred cell dead-end filtration unit (Sterlitech Corporation HP4750, Auburn, WA, USA), with a maximum volume of 316 mL, a membrane diameter of 47 mm, and an active membrane area of 8.55 cm^2^. The operating pressures were supplied by a compressor and were chosen between 3 and 6 bar, depending on the resistance of the membranes used. During the experiment, the pressure was measured and kept constant using a pressure gauge and further adjusted by the control valve. A homogeneous feed to the membrane was guaranteed by a stir-bar hanging above the membrane, while the filtration unit was placed on a magnetic stirrer set to 50 rpm. The permeate was collected in a beaker placed on an electronic balance (PCB 1000, Kern & Sohn GmbH, Balingen, Germany), connected to a data acquisition system, and the mass was recorded at regular 10 s intervals. The filtering continued until all the volume was filtered. The monitored mass flow was converted to volume flow using density at the given temperature. When a new filtration experiment was started, the first 5 mL of filtrate were discarded, considering a dead volume on the filtrate side of the filtration unit, and the tubes to the beaker collecting the filtrate. After filtration, the flange at the top of the filter was opened to remove the filter paper with the cake. The mass of the filtrate, filtration time, pressure, and filtration temperature were recorded for the calculation of a filtering rate.

#### 2.2.2. Membranes

The separation experiments were performed using the following membranes:

- Pall^®^ MF membrane (FluoroTrans W PVDF, Pall, Chardon, OH, USA) with a nominal pore size of 0.2 µm;
- TriSep polyethersulfone UF membrane with a MWCO of ~5000 Da (TriSep flat sheet membrane, UF5, PES, 47 mm, Sterlitech, Auburn, WA, USA);
- TriSep poly piperazine-amide NF membrane with MWCO ~200 Da (TriSep flat sheet membrane, TS80, PA, 47 mm, Sterlitech, Auburn, USA);
- TriSep polyamide-TFC RO-membrane (MWCO not available) (TriSep flat sheet membrane, ACM4, PA, 47 mm, Sterlitech, Auburn, USA).

Before the filtration experiments, the membranes were soaked in DI water for at least 24 h to ensure that any shipping or storage preservatives were washed out properly, and the membranes were allowed to swell. After a membrane had been placed in the stirred cell, compaction was guaranteed by filtering 20 mL (or until constant flux had been achieved) of deionized water at the chosen transmembrane pressure (TMP).

#### 2.2.3. Reference Membrane Flux Using Deionized Water

The permeabilities of all membranes were determined from the filtration of deionized water at a pressure of 3 bar, and after a stable flux had been achieved. The permeabilities obtained from these tests are referred to as the “clean membrane flux”. They are used as the benchmark to judge the impact of particulate and dissolved substances and the effect on membrane fouling.

#### 2.2.4. Filtration Operating Performance

**Figure 2:**
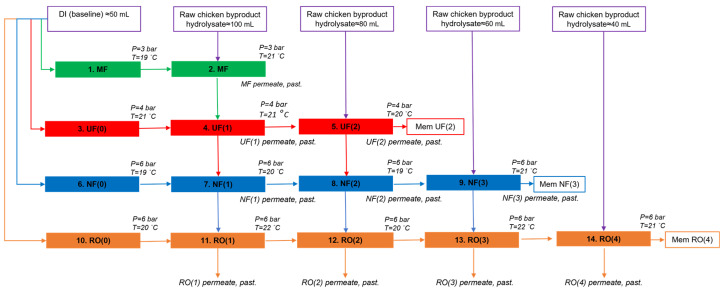
Workflow of the dead-end membrane filtration experiments treating chicken byproduct hydrolysate (P = pressure; T = temperature; past. = pasteurized).

The performance of four different membranes (MF, UF, NF, and RO) in the treatment of chicken hydrolysate was studied. The experimental flow is depicted in Figure 2. These processes yielded the samples MF, UF(1), UF(2), NF(1), NF(2), and NF(3), as well as RO(1), RO(2), RO(3), and RO(4), which were screened for inhibition of DPP-IV enzyme, and analyzed using size-exclusion chromatography and LC–OCD–OND. After each filtration step, samples of 7 mL permeate were reserved for further analysis of bioactivity. 

#### 2.2.5. Pasteurization of Permeates

At the end of each filtration step, the permeates produced were pasteurized at 90 °C for 30 min to avoid degradation of permeates caused by microorganisms. Pasteurization was achieved using a heating circulator with a capacity of 4.5 L (Julabo GmbH Seelbach, Germany).

#### 2.2.6. Calculation of Permeability

The mass flow rate obtained using the mass on the balance per unit of time was converted to a volume flow rate considering the density of the liquid at the given temperature and membrane flux J_m_ was calculated according to Equation (1).

(1) $$J_{m}=\frac{Q}{a}$$

where J_m_ = volumetric water flux through membrane [L/m^2^/h]; Q = flow rate [L/h]; a = membrane area [m^2^].

As the membrane flux is dependent on viscosity and, thus, on temperature, membrane flux J_s_, normalized for the standard temperature of 20 °C, was calculated according to Equation (2) [33].

(2) $$J_{s}=Jm_{\;}(\frac{\mu_{m}}{{µ}_{s}})$$

where J_s_ = flux at standard temperature (typically 20 °C) [L/m^2^/h]; J_m_ = flux at measured temperature [L/m^2^/h]; µ_m_ = dynamic viscosity of permeate at measured temperature [kg/m/s]; µ_s_ = dynamic viscosity of permeate at standard temperature [kg/m/s].

Further, the specific flux was normalized for pressure by calculating the permeability P_s_ at the standardized temperature of 20 °C according to Equation (3).

(3) $$P_{s}=\frac{J_{s}}{TMP}$$

where TMP = transmembrane pressure [bar].

### 2.3. Analytical Methods

#### 2.3.1. Turbidity, Conductivity, pH

The turbidity of filtrates at different stages of the filtration process was monitored and measured using a turbidimeter (Hach 2100Q, Loveland, CO, USA) with a repeatability of 0.01 NTU. The pH of the permeates was measured with a pH meter (Multi 7430, VWR, Ville Mont-Royal, QC, Canada) equipped with an electrode compensating for temperature variation. The conductivity of permeates was measured using a conductivity meter (Multi7430, VWR, Canada) with a measurement range of 0.00 to 20.00 mS/cm.

#### 2.3.2. Particle Size Distribution

Particle size concentration and distribution of raw chicken byproduct hydrolysate was measured using a Particle Counter PCSS fluid lite equipped with a LDS45/50 laser sensor (Markus Klotz GmbH, PCSS fluid lite, Bad Liebenzell, Germany) set to a particle size measuring range from 0.8 to 100 µm with 16 size classes, and a flow rate of 30 mL/min. During the measurement, the feed was stirred using a magnetic stirrer at 200 rpm at room temperature for all the experiments. A total of 10 mL of the raw chicken hydrolysate was measured in 5 replicates. Measured data were evaluated using the Protrend software, version SA1800-2.

#### 2.3.3. DPP-IV Inhibition Assay

Dipeptidyl peptidase IV (DPP-IV) inhibition study was performed using a commercial screening Assay Kit (ab133081) (Abcam PLC, Cambridge, UK). The assay is based on a release of the fluorescent moiety from the fluorogenic substrate (Gly-Pro-Aminomethylcoumarin or AMC), caused by the activity of the DPP-IV enzyme. For the initial screening, hydrolysates were tested using final concentrations of 1.0 mg/mL and 0.5 mg/mL. For IC_50_ value measurements, a total of nine concentrations were tested in a range from 0.01 mg/mL to 7.00 mg/mL, in triplicates. Inhibitor samples were prepared by dissolving lyophilized hydrolysates in the assay buffer (20 mM Tris-HCl, pH 8.0, containing 100 mM NaCl, and 1 mM EDTA) to the corresponding stock solution and subsequently filtering them through a Millex-HV PVDF 0.45 μm 33 mm filter (MilliporeSigma, Burlington, MA, USA). The assay kit was used according to the instructions from the manufacturer. Experiments were performed in triplicates, in 96-well microplates, with a final volume of 100 µL per well. Inhibitor wells contained 10 µL of the test sample, 10 µL of the enzyme solution, 50 µL of the substrate solution, and 30 µL of the assay buffer. To the initial activity wells, assay buffer was added instead of inhibitor solution, and to the background wells, assay buffer was added instead of both inhibitor and the enzyme solution. Sitagliptin (final conc. = 100 µM) was used as a positive control for the inhibition. The assay mixture was incubated for 30 min at 37 °C before acquiring emission values (λ_exc_ = 355 nm, λ_em_ = 455 nm). Fluorescence measurement was carried out using a Synergy H1 hybrid multi-mode microplate reader (SynergyBiotek, Winooski, VT, USA). The average value of background fluorescence was subtracted from all the readings of the samples. The % inhibition of each sample was calculated relative to the initial activity of the enzyme (with H1, out of any inhibitor added) as follows:

(4) $$\%\mathrm{Inhibition}=\frac{\mathrm{Initial}\;\mathrm{enzyme}\;\mathrm{activity}\;-\;\mathrm{Enzyme}\;\mathrm{activity}\;\mathrm{with}\;\mathrm{inhibitor}}{\mathrm{Initial}\;\mathrm{enzyme}\;\mathrm{activity}}\times \;100\;$$

#### 2.3.4. Size-Exclusion Chromatography

Samples of permeates for high-sensitivity, size-exclusion chromatography with organic carbon detection and organic nitrogen detection (LC–OCD–OND) analysis were collected in dedicated 10 mL oven-burned DOC-free amber glass vials. For the analysis, samples were diluted 400 times using deionized ultrapure water. Signals resulting from the deionized water were subtracted from the signals obtained from diluted samples before further calculation of the fraction’s concentrations. Quantitative analysis of organic matter fractions was performed by a modified version of the LC-OCD-8 model; SC2000 system (Postnova analytics, Landsberg, Germany) together with high-sensitivity organic carbon detection OCD (DOC-Labor Huber, Karlsruhe, Germany), UV-detector PN3211 (Postnova analytics) and SEC column filled with Toyopearl HW-50S (30 µm, dimensions 250 × 20 mm) [34,35]. Data interpretation was carried out with the evaluation software ChromCALC, version 2.4.

In addition, the size-exclusion chromatography equipped with UV detection (LC–UV) was performed as described by Wubshet et al. [36]. Chromatographic runs were controlled from Chromeleon™ Chromatography Data System (CDS) software version 6.8 (Thermo Fisher Scientific, Waltham, MA, USA). From chromatographic runs of both molecular weight standards and test hydrolysates, a UV trace of 214 nm was used. SEC chromatograms of the hydrolysates were converted to molecular weight distributions and weight average molecular weights (MW) were calculated, using a calibration curve constructed based on molecular weight standards (Table 1). These calculations were performed in MATLAB (R2022b, The Mathworks Inc., Torrance, CA, USA), using the openly available SEC2MWD toolbox [37]. Areas of the specified fractions in the chromatogram were calculated using the same toolbox.

### 2.4. Statistical Analysis

All data are presented as means with standard deviations.

## 3. Results and Discussion

### 3.1. Physical and Chemical Characterization of Hydrolysate

The physical and chemical properties of raw (unfiltered) and MF-filtered hydrolysates are given in Table 2. The pH of the hydrolysates might impact the membranes, however, in the measured samples the pH was neutral. Conductivity reflects the concentration of salts. High salt concentrations will cause high osmotic pressure in NF and RO. Conductivity was moderately low with 600 to 800 µS cm^−^^1^. Turbidity is a proxy for particle concentrations and allows prediction of how particle concentrations might deposit on the membranes and reduce flux. The measured turbidity of the unfiltered hydrolysate was approximately 33 NTU. MF (0.2 µm pore size) removed most of the particulate matter, decreasing the turbidity from 33.6 to 5.8 NTU. Pasteurization of the hydrolysate increased the conductivity for both the unfiltered and filtered samples. This points to the dissolution of otherwise colloidal substances, which even pass the MF membrane, at elevated temperatures during pasteurization. This hypothesis was confirmed by the decrease in turbidity of the filtered hydrolysate from 5.8 NTU to 4.1 NTU, due to pasteurization. For the unfiltered samples, the dissolution of relatively few colloidal particles was not reflected in a measurable lower turbidity.

### 3.2. Particle Size Distribution

**Figure 3:**
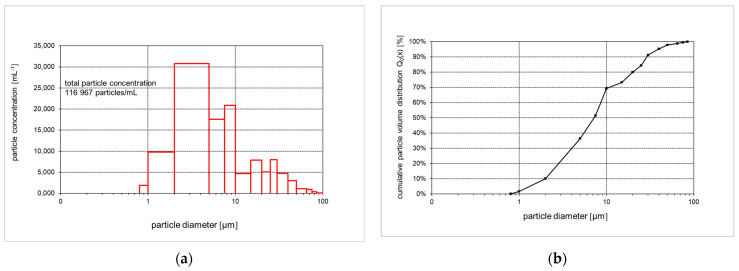
Particle size distribution: (**a**) particle concentration of the raw unfiltered chicken byproduct hydrolysate (number of particles per mL); (**b**) cumulative particle size distribution of the raw unfiltered chicken byproduct hydrolysate.

Figure 3 shows the particle concentrations in the different size classes (a) and the particle size distribution (b) of the raw unfiltered chicken byproduct hydrolysate. The total particle concentration in the raw hydrolysate was found to be 116,967 particles per mL (Figure 3a). This is a high concentration and corresponds to the high turbidity. Diameters of 0.8 µm are below the optical resolution of the instrument, and, therefore, not counted. Most particles were found in the size classes below 10 µm, which comprise about 70% of all particles. Also, a break in the slope of the cumulative number distribution can be seen, as the slope decreases sharply at that size (Figure 3b). In the production of the hydrolysate (Section 2.1) depth filters were used, which, according to the manufacturer, perform best at particle sizes above 15 µm, i.e., remove particles above 15 µm best. Thus, the results from the particle size measurements were in accordance with the production process. The high particle concentration and the size distribution mainly demonstrate that most of the particles were below 10 µm in diameter. This confirms that high amounts of particles will deposit on the membranes when using UF, NF, and RO for the separation of dissolved substances.

### 3.3. Effect of Membrane Filtration on Concentrations of Organic Matter Fractions

**Figure 4:**
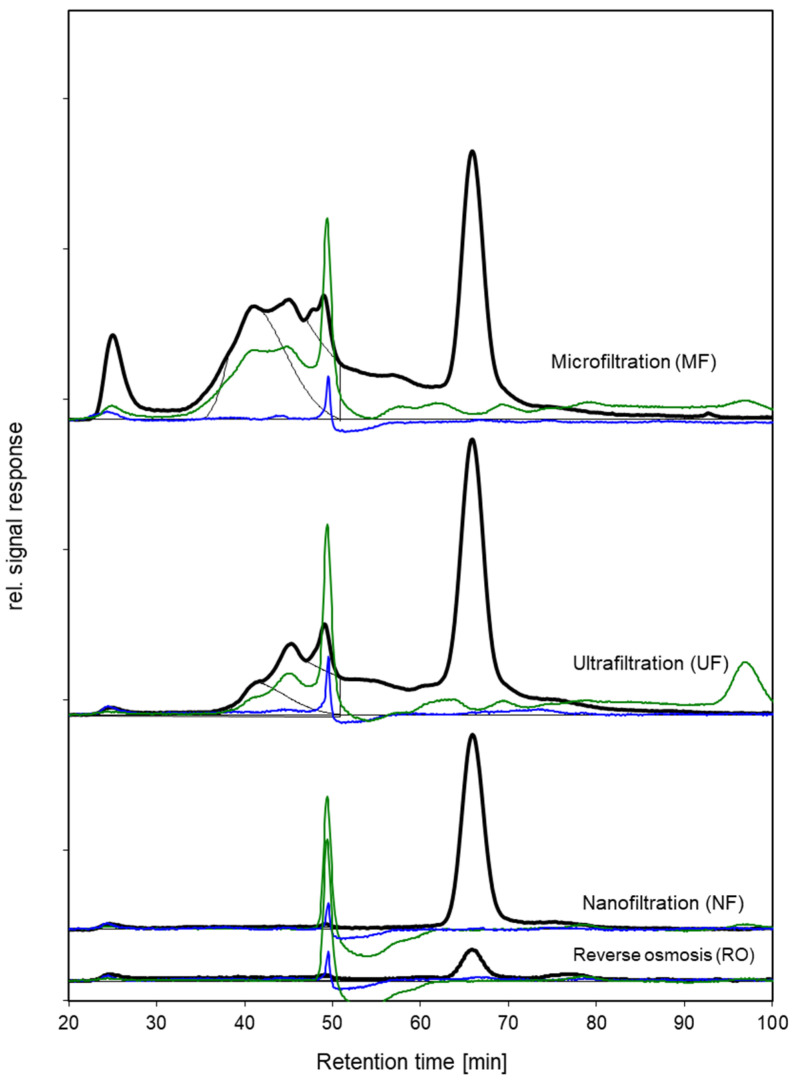
LC–OCD chromatograms for filtered chicken byproduct hydrolysate using MF, UF, NF, and RO membranes. Carbon signals (black line), nitrogen signals (green line), UV 254 absorption signals (blue line), natural organic matter (gray line).

Concentrations of dissolved organic substances in the hydrolysate were analyzed using LC–OCD–OND. Figure 4 shows the chromatograms with carbon, nitrogen, and UV signals for chicken byproduct hydrolysate that had been filtered using MF, UF, NF, and RO membranes. The chromatographic organic carbon (CDOC) is illustrated as distinctive peaks for specific molecular weight fractions. The size of the fractions was obtained and reported specifically for carbon and nitrogen by integrating the signal response over time. Fraction A mainly contains high-molecular-weight compounds with a MW > 20 kDa, while fraction B has organic matter of about 1 kDa, fraction C of MW > 500 Da, and fraction D organic substances of MW < 500 Da. As low-molecular-weight compounds, different substances with MW < 500 Da eluted at approximately the same time as fraction D (they were reported together with fraction D). The low-molecular-weight substances (LMW) of fraction D were eluted after 55 min and up to 100 min. A specific peak appeared at 66 min and was observed for all four filtrations of the chicken byproduct hydrolysate (Figure 4).

The concentrations of dissolved organic carbon (DOC), and C and N in the different fractions, are compiled in Table 3. It is important to note the ratio N/C in the hydrolysate after MF. This ratio indicates the contribution of proteinaceous compounds to the organic substances. For fraction A (MW > 20 kDa), nitrogen was found in a concentration of 16 ppm in the hydrolysate. Based on a rule of thumb for the carbon/nitrogen mass ratio of three in proteins and almost no nitrogen in polysaccharides [34], the percentage of proteinaceous carbon in fraction A can be estimated to be 43%. For fraction B, this was estimated to be 91%, and for fraction C, it was 64%. Fraction D may contain small peptides, single amino acids, and other low-molecular-weight compounds with MW > 500 Da. Therefore, a calculation of the percentage of proteinaceous carbon was not reasonable for fraction D. Considering DOC, i.e., the sum of all fractions, the membranes with decreasing pore sizes from MF, UF, and NF to RO removed an increasing amount of dissolved organic substances. While DOC was about 2.1 g (2134 ppm C) in the MF-filtered hydrolysate, the concentration decreased to about 1.7 g after UF, to 0.39 g after NF, and further to 0.18 g after RO. Hydrophobic organic carbon (HOC), which contains organics that irreversibly stick to the SEC gel, eventually passed the UF membrane, while both NF and RO decreased to 70 to 80 ppm.

Fraction A, which contains high-molecular-weight substances such as proteins with MW > 20 kDa, was removed completely by UF. While the hydrolysate contains 114 ppm of fraction A, the concentration was decreased below the limit of quantification (LOQ) by UF. Also, fraction B, which contains organic matter in the molecular weight size range of 1 kDa, was removed by more than 75% (325 ppm past MF, 76 ppm past UF). Fraction C was removed to a minor extent by UF (about 26%, from 178 ppm to 132 ppm). Thus, it might happen that some of the desired peptides were removed by UF as well. Together, the fractions A and B comprised about 34% of the CDOC and about 20% of the DOC. Consequently, as the desired peptides are expected in the molecular size range of 500 Da, applying UF may remove about 20% of the undesired organic carbon. NF and RO removed both fractions A and B almost completely (>97%). However, they also removed fraction C, with MW around 500 Da, completely, indicating that they also may remove the desired peptides. Fraction D with MW < 500 kDa passed UF almost completely, while it was removed completely by NF and RO. The same applies to the removal of low-molecular-weight neutrals by UF, which passed UF completely. About 50% passed NF, and about 13% even passed RO. Accordingly, as desired bioactive peptides have an average molecular weight of 514 Da [8], it can be concluded that UF may be a feasible separation to remove undesired high-molecular-weight substances from the chicken hydrolysate. Based on the LC–OCD analysis discussed above, the sought bioactive peptides were eluted as fractions C and D. Consequently, NF and RO would retain these bioactive peptides in the concentrate, together with all other organic substances in the same MW range, as well as those with a higher molecular weight (fractions A and B). NF and RO would, therefore, concentrate all types of organic substances. Hence, these two membrane filtrations were ruled out as a potential downstream separation approach for up-concentration of the sought bioactive peptides (i.e., fraction C). However, UF was shown to retain high-molecular-weight substances in fractions A and B, while the bioactive peptides in LC–OCD fractions C and D were eluted (Figure 4). Based on this, it is expected that the number of bioactive compounds will be higher in UF-filtered hydrolysate, compared to MF-filtered hydrolysate. Therefore, UF was selected as a promising filtration approach for further evaluation of bioactivity and molecular weight distribution of the permeates.

### 3.4. Membrane Permeabilities

**Figure 5:**
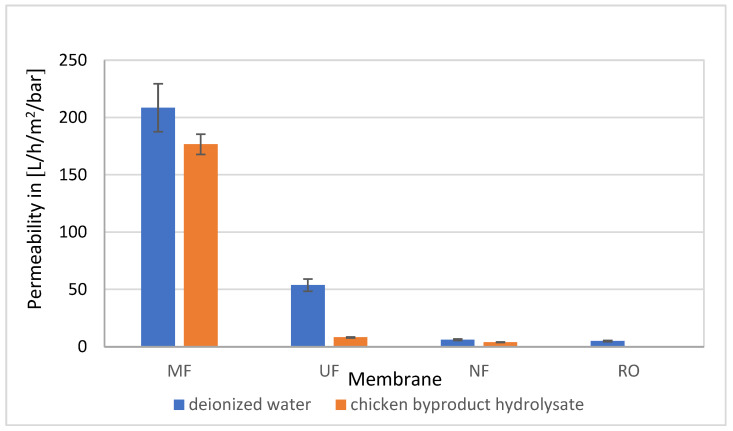
Membrane permeabilities when filtering deionized water and chicken byproduct hydrolysate with MF, UF, NF, and RO membranes.

Figure 5 shows the permeabilities of the membranes when filtering deionized water and raw chicken byproduct hydrolysate through the MF, UF, NF, and RO membranes. The permeabilities of the deionized water were in the range expected according to the manufacturer’s specifications. While the permeability for the UF membrane was about 25% of the MF’s permeability, the NF’s permeability was about 3% (6.2 L/m^2^/h/bar), and the RO membrane permeability was about 2% (4.6 L/m^2^/h/bar) of the MF membrane’s permeability. As expected, when chicken byproduct hydrolysate was filtered, permeabilities were lower than the permeabilities of deionized water. The MF mainly removed particles with a diameter larger than about 0.2 µm and the permeability decreased by 15%. However, the UF decreased by about 85% (8.2 compared to 53.7 L/m^2^/h/bar) and the NF by about 35% (4.0 compared to 6.2 L/m^2^/h/bar). With RO, the permeability was so low that it was not measurable. During several hours, only a few milliliters of sample were produced. The decreases in permeabilities can be compared to the removal of organic carbon by the respective membrane filtration since the relative permeability, when compared to the permeability with deionized water, reflects the relative number of substances removed from the chicken byproduct hydrolysate and their properties in the respective pore size range. While MF removes particles, dissolved organic carbon (DOC) was not impacted. However, the high-molecular-weight substances from fractions A and B were removed almost completely by UF sticking to the membrane and causing a pronounced decrease in permeability (Table 3). Furthermore, the removal of DOC by NF was even more pronounced than for UF. As the high-molecular-weight compounds had been removed before by UF. these did not block the NF membrane. The properties of the compounds in fractions C and D removed by NF did not block the membrane to an extent as high as the high-molecular-weight substances in fractions A and B. Hence, with moderate permeability and the desired molecular weight selectivity discussed previously, UF was chosen as a potential approach for the up-concentration of bioactive peptide fractions from crude chicken byproduct hydrolysate.

**Figure 6:**
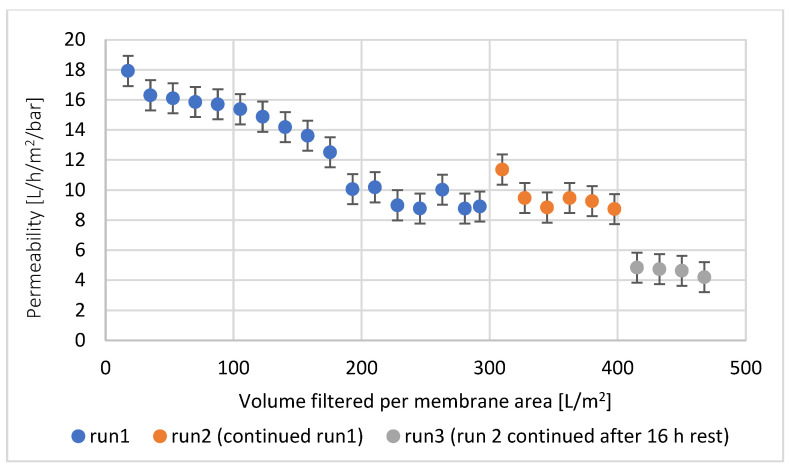
Permeability during ultrafiltration of chicken byproduct hydrolysate as a function of filtered volume.

Figure 6 shows the impact of the treated volume on permeability, due to the deposit of particles and high-molecular-weight substances on the UF membrane. Membrane filtration was carried out in three phases. First, 250 mL (300 L/m^2^) was filtered without interruption. Then, the filtration was stopped and was taken into operation after refilling of hydrolysate for filtration. Last, the filtration was stopped for 16 h, to check the effect of an interruption of operation. For the first 300 L/m^2^, permeability decreased continuously, due to deposition of particles and high-molecular-weight substances. It reached a plateau due to no further high-molecular-weight substances being able to penetrate and block the pores, due to lack of space and large enough pore openings. Further organic substances were deposited mainly on top of the membrane. Resting of the deposit, which might happen during an interruption of the process, may cause biological changes due to bacterial growth leading to higher resistance and, thus, lower permeability [20].

### 3.5. Effect of Applied Downstream Processing on the DPP-IV Inhibitory Activity of the Chicken Byproduct Hydrolysate

**Figure 7:**
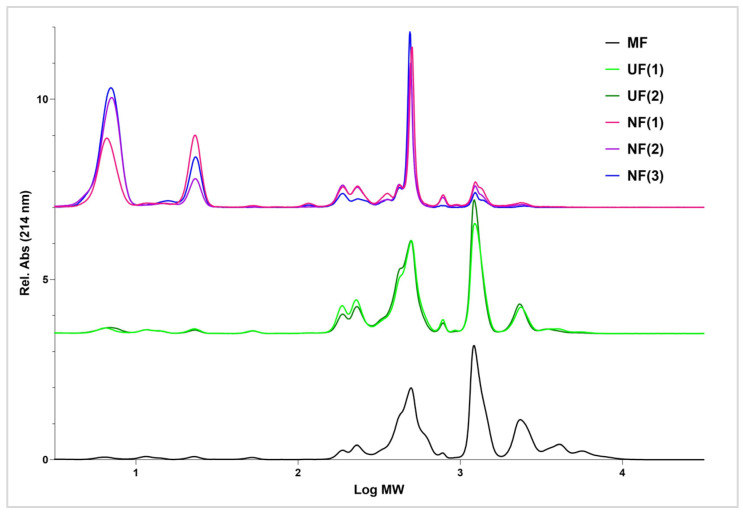
Size-exclusion chromatograms of permeates MF, UF(1), UF(2), NF(1), NF(2), and NF(3); (c = 10 mg/mL, V (injection) = 10 µL).

The effect of the performed downstream processing on the DPP-IV inhibitory activity of MDCR hydrolysate was evaluated. Bioactivity (Table 4) was correlated with molecular weight distribution profiles and average molecular weights (AMW) (Table 5, Figure 7).

**Figure 8:**
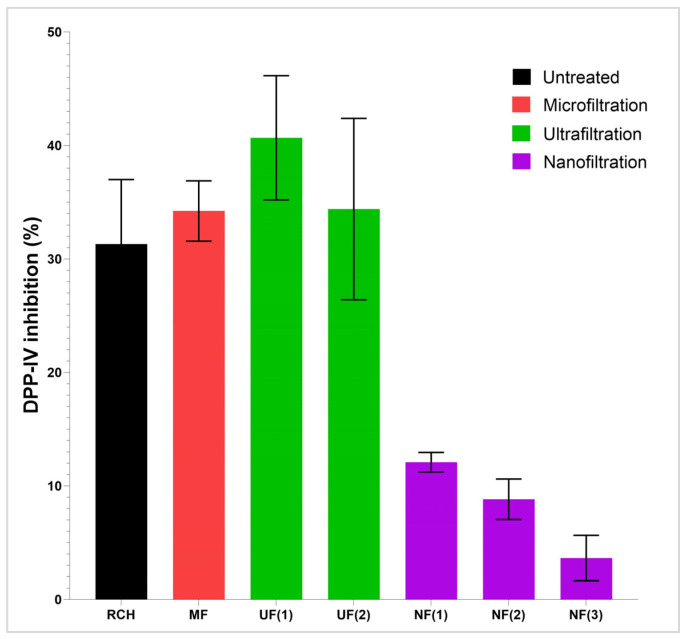
DPP-IV inhibition (%) of raw chicken byproduct hydrolysate (RCH) and the corresponding permeates from MF, UF(1), UF(2), NF(1), NF(2), and NF(3). All samples were tested at a concentration of 0.5 mg/mL.

The preliminary screening involved the permeates produced as described in Section 2.2.4. The permeates MF, UF(1), UF(2), NF(1), NF(2), and NF(3) were tested at the assaying concentrations of 0.5 mg/mL (Figure 8, Table 5 and Table 6). Comparing all the treatments, hydrolysates processed only by microfiltration and ultrafiltration (MF, UF(1), and UF(2)), show the best DPP-IV inhibition (59.5–60.0% at 1 mg/mL and 34.2–40.7% at 0.5 mg/mL). These samples show dose-responsive behavior. This agrees with hypotheses discussed in Section 3.3, as well as with reports in the literature that confirm that the bioactive peptides usually comprise two to twenty amino acids [38,39]. The food-derived peptides reported in this review are those mainly utilized in the food and medical fields, with the ability to exert antimicrobial, antidiabetic, and antioxidant bioactivities, among others [38].

The nanofiltration process notably decreases the inhibitory activity, and these permeates (NF(1), NF(2), and NF(3)) show low DPP-IV inhibition (9.5–21.8% at 1 mg/mL and 3.6–12.1% at 0.5 mg/mL). This confirms the conclusions drawn from the LC–OCD–OND analysis, that NF retains a major part of the bioactive peptides, while they pass the UF membrane. Samples obtained by RO treatments were not evaluated due to very low material recovery, which rendered this treatment not feasible for the intended purpose.

**Figure 9:**
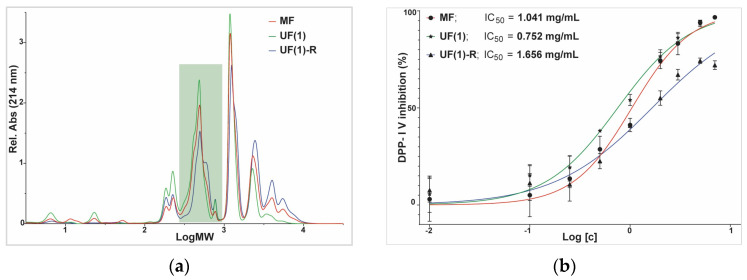
Size-exclusion chromatogram: (**a**) size-exclusion chromatogram of the chicken byproduct hydrolysate processed by MF (red line), UF (green line), and retentate from UF (blue line); c = 10 mg/mL, V (injection) = 10 µL, marked with a green rectangle is the fraction containing the bioactive peptides; (**b**) dose-response curves of DPP-IV inhibition for the chicken byproduct hydrolysate processed by MF (red line), UF (green line), and retentate from UF (blue line); µF—microfiltration (MF).

To study the effect of the promising MF and UF permeates more thoroughly, IC_50_ values of these samples were determined (Figure 9). In addition, a portion of a retentate (UF(1)-R) from the production of sample UF(1) was collected, and its IC_50_ value was determined as well for comparison purposes. Ultrafiltration sample UF(1) showed an IC_50_ value of 0.75 ± 0.07 mg/mL, comparable to other potent DPP-IV inhibitors derived from various food sources [41], and significantly more potent compared to microfiltration sample MF, which showed an IC_50_ value of 1.04 ± 0.12 mg/mL. On the contrary, the retentate sample UF(1)-R showed a significantly lower potency at an IC_50_ value of 1.66 ± 0.19 mg/mL.

The statistical analysis results of the confidential interval of IC50 of the MF and UF permeate samples illustrated in Figure 9 are shown in Table 7.

SEC chromatograms (Figure 9a) distinctly show the decrease in the amount of the largest peptides, with molecular weights larger than 1785 g/mol. The average molecular weight, calculated from the SEC chromatograms, was 883 g/mol for the UF(1), and 1437 g/mol for MF (0.2 µm). Retentate (UF(1)-R) had an AMW of 1759 g/mol. The amount of the fraction with the largest molecules decreased from 24.7% in 0.2 µm permeate to 10.6% in UF(1) permeate (Table 4). The UF permeates had the largest amount of the fraction with molecular weights from 283 to 906 g/mol, corresponding to fractions C and D from LC–OCD–OND measurements. Food-derived bioactive peptides offer much promise in improving human health, while providing a valuable resource for food producers to prepare value-added products and better utilize the often-wasted by-products. A better understanding of manufacturing, consumer-related, and regulatory aspects would enable broader acceptance and faster utilization of their potential [40,41].

To conclude, the use of UF and NF in the fractionation and concentration of peptides is not new, but is gradually increasing. The use of UF membranes seems to be adapted to the separation of chicken-byproduct-based peptides. Polysaccharides appear to be one of the main compounds separated along with proteins, but new studies should also focus on detailed separation of chicken-hydrolysate-based peptides from various acids, which may limit application of food-based products, and minerals, which can be in higher amounts when compared to other protein hydrolysates. Membrane technology application in the downstream of food products requires more deep studies to define ways to improve it. The impact and choice of operating parameters is still not clear and requires further understanding. For chicken byproduct hydrolysates, membrane processes are still mainly used as an analytical tool, and the separation process is not explored in terms of process parameters. Mass transfer, concentration polarization and fouling phenomena, effect of raw material characteristics (composition, pH, ionic strength, concentration), and interactions between the membrane material and chicken byproduct hydrolysate are little reported in the literature. Operational costs are also a very relevant concern in the processing of chicken-based byproducts and waste streams. The separation and fractionation of chicken-based protein hydrolysates in fewer steps or in higher concentrations is desired, for economic reasons. In fact, production of chicken byproduct hydrolysates using different technologies such as cross-flow separation could be a possibility to be explored as well. Further studies are still needed to improve and extend the scope of membrane technology for an efficient and cost-effective production of peptides from chicken byproduct hydrolysates.

## 4. Conclusions

The advancement and development of technology have facilitated the research and utilization of bioactive peptides. The availability of peptide-based products such as therapeutic drugs, functional foods, and additives in the modern market is a vivid outcome of the tremendous progress made. Despite their limitations, bioactive peptides have great potential. However, more studies need to be conducted since the outlined strategies have limitations also. The application of membrane separation technologies on the recovery of value-added compounds such as bioactive peptides from chicken byproduct depends on the development of integrated processes adapted to the specificities of this materials. Peptide and protein fractionation should consider the associated effects of feed, membrane, and processing parameters so that maximum membrane performance is achieved, and an economically viable recovery of peptides is possible.

This study presents the possibility of UF/NF separation as a downstream processing for chicken byproduct hydrolysates. The particle size distribution measurements of the chicken byproduct hydrolysate show that most of the particles are below 10 µm in diameter, confirming that high amounts of particles will deposit on the membranes when using UF, NF, and RO for the separation of dissolved substances. Of the four membranes studied, the UF membrane shows the best separation properties with respect to maximizing the yield and up-concentration of the bioactive peptides. It became clear that the membranes with decreasing pore sizes from MF, UF, and NF to RO removed an increasing amount of dissolved organic substances. The LC–OCD analysis confirms that NF and RO retain the bioactive peptides in the concentrate, together with all other organic substances in the same MW range, as well as those with a higher molecular weight. Hence, these two membrane filtrations were ruled out as a potential downstream separation approach for the up-concentration of the sought bioactive peptides. The permeabilities of the filtered chicken byproduct hydrolysates were considerably lower than the permeability of deionized water. The MF mainly removed particles with a diameter larger than about 0.2 µm and the permeability decreased by 15%. However, the permeability of UF decreased by about 85% and the permeability of NF by about 35%. The permeability of the RO was so low that it was not measurable. With moderate permeability and the desired molecular weight selectivity, UF was chosen as a potential approach for the recovery of bioactive peptide fractions from crude chicken byproduct hydrolysate. The permeate from UF showed the IC_50_ value of 0.75 mg/mL, significantly more potent compared to microfiltration permeate from MF (IC_50_ 1.04 mg/mL). SEC chromatograms show a decrease in the amount of the largest peptides.

In conclusion, UF was demonstrated to be a feasible downstream process for up-concentration of small-molecular-weight bioactive peptides from chicken byproduct hydrolysate. Furthermore, the obtained scientific results are considered as a preliminary experimental result, and more scientific work on the UF membranes with different grade (MWCO) is needed and is already designed in the second scientific article as a continuation of this present work. Addressing specific challenges and discovering new bioactive peptides brightens the future for bioactive peptides, which could result in extensive utilization and huge market demand.

## Figures and Tables

**Table 1:** DPP-IV inhibition (%) by permeates MF, UF(1), UF(2), NF(1), NF(2), and NF(3); c = 1 mg/mL.

Sample	DPP-IV Inhibition (%)	St. Deviation (%)	N (Samples)
RCH *	59.78	6.43	4
MF	60.05	6.01	4
UF(1)	59.46	0.64	4
UF(2)	59.85	7.51	3
NF(1)	21.80	5.52	3
NF(2)	15.93	1.05	4
NF(3)	9.49	1.42	4

* RCH = raw chicken hydrolysate.

**Table 2:** Physical and chemical properties of chicken byproduct hydrolysate.

Sample	pH Mean (SD)	Turbidity (NTU) Mean (SD)	Conductivity (µS cm^−1^) at 20 °C Mean (SD)
Raw (unfiltered) chicken byproduct hydrolysate	7.2 (0.08)	33.6 (0.39)	618.0 (0.69)
Raw (unfiltered) chicken byproduct hydrolysate—pasteurized	6.9 (0.12)	33.7 (0.21)	719.0 (1.81)
Filtered (MF) chicken byproduct hydrolysate	7.1 (0.12)	5.8 (0.57)	621.5 (1.76)
Filtered (MF) chicken byproduct hydrolysate—pasteurized	7.0 (0.08)	4.1 (0.45)	838.3 (1.36)

**Table 3:** LC–OCD results of chicken byproduct hydrolysate dead-end filtration.

Organic Matter			Filtration			
			Microfiltration (MF-2.) Mean (SD)	Ultrafiltration (UF-4.) Mean (SD)	Nanofiltration (NF-11.) Mean (SD)	Reverse Osmosis (RO-11.) Mean (SD)
DOC [ppm-C]	DOC * [ppm-C]		2134	1674	387	182
	HOC * [ppm-C]		852 (0.23)	819 (0.19)	70 (0.16)	81 (0.21)
	CDOC * [ppm-C]		1282 (1.05)	855 (0.96)	317 (1.32)	101 (1.00)
NOM	A > 20 kDa	DOC * [ppm-C]	114 (1.69)	<2	<2	<2
		DON * [ppm-N]	16 (0.56)	4 (0.74)	4 (0.61)	4 (0.62)
		N/C * [µg/µg]	0.14 (0.22)	-	-	-
		Proteins [%]	43 (1.31)	-	-	-
	B~1 kDa	DOC * [ppm-C]	325 (1.04)	76 (1.12)	7 (0.84)	8 (0.78)
		DON * [ppm-N]	98 (0.32)	20 (0.76)	<2	<2
		N/C * [µg/µg]	0.30 (1.54)	0.26 (0.84)	0.12 (0.96)	0.07 (0.92)
		Proteins [%]	91 (0.21)	78 (0.33)	-	-
	C~500 Da	DOC * [ppm-C]	178 (0.15)	132 (0.17)	6 (0.09)	7 (0.06)
		DON * [ppm-N]	38 (0.15)	33 (0.21)	0 (0.00)	0 (0.00)
		N/C * [µg/µg]	0.21 (0.23)	0.25 (0.32)	0.00 (0.00)	0.00 (0.00)
		Proteins [%]	64 (0.46)	75 (0.30)	-	-
	D < 500 Da	DOC * [ppm-C]	35 (0.83)	29 (0.52)	<2	<2
		DON * [ppm-N]	44 (0.66)	38 (0.51)	24	26
		N/C * [µg/µg]	1.26 (0.07)	1.33 (0.08)	-	-
		Proteins [%]	-	-	-	-
	LMW	Neutrals [ppm-C]	630 (0.12)	617 (0.10)	302 (0.14)	84 (0.22)

* DOC: dissolved organic carbon; HOC: hydrophobic organic carbon; CDOC: chromatographic organic carbon; DON: dissolved organic nitrogen; NOM: natural organic matter; LMW: low molecular weight; N/C: nitrogen/carbon; A, B, C, D: fractions based on MW.

**Table 4:** DPP-IV inhibition (%) by permeates MF, UF(1), UF(2), NF(1), NF(2) and NF(3); c = 0.5 mg/mL.

Sample	DPP-IV Inhibition (%)	St. Deviation (%)	N (Samples)
RCH *	31.31	5.68	4
MF	34.22	2.65	4
UF(1)	40.68	5.48	4
UF(2)	34.40	8.00	3
NF(1)	12.09	0.87	2
NF(2)	8.82	1.78	4
NF(3)	3.65	2.00	4

* RCH = raw chicken hydrolysate.

**Table 5:** Weight average molecular weights (AMW) of permeates RCH, MF, UF(1), UF(1)-R, UF(2), NF(1), NF(2) and NF(3) and area sizes of specific fractions in the chromatogram (% of the total chroma-togram area); fraction 1 (>1785 g/mol; r.t. 5–7.85 min), fraction 2 (906–1785 g/mol; r.t. 7.85–8.70 min), fraction 3 (283–906 g/mol; r.t. 8.70–10.13 min) and fraction 4 (<283 g/mol; r.t. 10.13–20 min).

Sample	AMW (g/mol)	Fraction 1 (%)	Fraction 2 (%)	Fraction 3 (%)	Fraction 4 (%)
RCH *	1417.19	24.67	30.76	33.34	11.24
MF	1437.44	24.71	30.78	33.58	10.94
UF(1)	883.35	10.58	28.09	39.67	21.66
UF(1)-R	1759.08	33.23	28.20	28.12	10.46
UF(2)	1069.66	10.45	30.28	40.02	19.24
NF(1)	479.20	2.47	6.94	27.76	62.83
NF(2)	659.48	1.81	5.13	23.32	69.75
NF(3)	185.73	0.33	3.14	24.62	71.91

* RCH = raw chicken hydrolysate.

**Table 6:** Retention times of standards (bovine serum albumin, albumin from chicken egg white, carbonic anhydrase from bovine erythrocytes, lysozyme, cytochrome c from bovine heart, insulin chain B oxidized from bovine pancreas, angiotensin II human, bradykinin fragment 1–7, [D-Ala2]-leucine encephalin, Val–Tyr–Val, and tryptophan) analyzed on BioSep2000 column for molecular weight calibration. Values from triplicate (i, j and k) measurements are presented.

	Molecules	MW (g/mol) ^a^	RT (i)	RT (j)	RT (k)	Mean RT	SD	LogM _W_
1	Bovine serum albumin	66,000	5.933	5.933	5.933	5.933	0.000	4.820
2	Albumin from chicken egg white	44,287	5.967	5.967	5.967	5.967	0.000	4.646
3	Carbonic anhydrase	29,000	5.975	5.975	5.975	5.975	0.000	4.462
4	Lysozyme	14,300	6.158	6.158	6.150	6.155	0.005	4.155
5	Cytochrome c from bovine heart	12,327	6.058	6.050	6.050	6.053	0.005	4.091
6	Insulin chain B oxidized from bovine pancreas	3496	7.408	7.417	7.417	7.414	0.005	3.544
7	Angiotensin II human	1046	7.600	7.608	7.608	7.605	0.005	3.020
8	Bradykinin Fragment 1–7	757	8.158	8.158	8.150	8.155	0.005	2.879
9	[D-Ala2]-Leucine enkephalin	570	9.817	9.825	9.833	9.825	0.008	2.756
10	Val–Tyr–Val	379	9.592	9.600	9.600	9.597	0.005	2.579
11	L-Tryptophan	204	10.433	10.442	10.442	10.439	0.005	2.310

^a^ Rounded to the nearest whole number. RT: retention time (min); MW: molecular weight (g/mol); SD: standard deviation.

**Table 7:** Statistical analysis results of the confidential interval of IC_50_.

Sample	95% Confidence Interval of IC_50_
MF	0.8963 to 1.186
UF(1)	0.6639 to 0.8410
UF(1)—R	1.429 to 1.883

## Data Availability

Data are contained within the article.
